# Body Image and Voluntary Gaze Behaviors towards Physique-Salient Images

**DOI:** 10.3390/ijerph18052549

**Published:** 2021-03-04

**Authors:** April Karlinsky, Holly Howe, Melissa de Jonge, Alan Kingstone, Catherine M. Sabiston, Timothy N. Welsh

**Affiliations:** 1 Department of Kinesiology, California State University, San Bernardino, CA 92407, USA; april.karlinsky@csusb.edu; 2 Fuqua School of Business, Duke University, Durham, NC 27708, USA; holly.howe@duke.edu; 3 Faculty of Kinesiology and Physical Education, University of Toronto, Toronto, ON M5S 2W6, Canada; melissa.dejonge@utoronto.ca (M.d.J.); catherine.sabiston@utoronto.ca (C.M.S.); 4 Department of Psychology, University of British Columbia, Vancouver, BC V6T 1Z4, Canada; alan.kingstone@ubc.ca

**Keywords:** affect, body appreciation, body envy, physical self-perceptions, media

## Abstract

The purpose of this study was to explore body image correlates of voluntary consumption of physique-salient media. A secondary aim was to assess changes in affect following media consumption. Young adult men (*n* = 47; mean age = 20.2 years) and women (*n* = 87; mean age = 19.5 years) were discretely exposed to images of same-sex models with idealized- and average-physiques while completing an irrelevant computer task. Voluntary gaze at the images was covertly recorded via hidden cameras. Participants also completed measures of affect before and after the computer task. Measures of body-related envy, body appreciation, and self-perceptions of attractiveness, thinness, and physical strength were completed. Men and women did not differ in how often nor for how long they looked at the images overall, but body image variables were differentially associated with their voluntary gaze behaviors. For men, higher body-related envy and lower body appreciation were correlated with more looks at the average-physique model. Although women reported higher body-related envy than men, envy and body appreciation were not significant correlates of gaze behaviors for women. Both men and women experienced a general affective decrease over time, but only for men was the change in negative affect associated with their time spent looking at the ideal-physique image. Overall, these findings suggest that body-related envy and body appreciation influence how men choose to consume physique-salient media, and that media consumption may have negative consequences for post-exposure affect. Body image factors appear to be more strongly associated with behavior in men, perhaps because men are generally less often exposed to physique-salient media and, in particular, to average-physique images.

## 1. Introduction

People are frequently exposed to media depicting the Western idealized physique—attractive, tall, thin women and muscular men [1,2]. Exposure to physique-salient images has increased with advancements in social media; it is estimated that 700 million new photos are uploaded to Facebook, Snapchat, and Instagram each day [3], with many of these images portraying idealized physiques [4]. Repeated exposure to media featuring idealized physiques can have a substantial negative impact on the mental and physical well-being of women [5] and men [1,6]. Specifically, body dissatisfaction, body anxiety, inactivity, disordered eating, depression, and low self-esteem are among the consequences of exposure to idealized physiques [1,5,7,8]. It is also likely that there are individual differences in the images that are chosen for viewing as well as individual differences in the affective responses to the consumption of those images. For example, based on social comparison theory [9], individuals high on appearance social comparison report more negative body image outcomes related to media exposure [10]. Further, according to self-objectification theory [11], internalization of the ideal body and body dissatisfaction may also influence the extent of negative outcomes related to exposure to physique-salient images [12]. The overall purpose of the present study was to gain a better understanding of the individual difference factors associated with the choice in viewing images with different body types and the affective responses to those choices.

There is compelling evidence that some individuals may be more susceptible than others to the negative consequences of consuming media containing idealized physiques (e.g., [6,13,14]). Body satisfaction is perhaps the most well-known moderator of the effects of idealized media exposure. These studies of body image typically employ an overt exposure methodology in which participants are presented with physique-salient stimuli (e.g., images, advertisements, commercials) either one at a time (such that participants are required to observe the presented stimulus) or within a matrix, with participants allowed to look between the presented images freely (for reviews, see [14,15,16]). Compared to individuals with high body satisfaction scores, young men [17] and women [18] who were more dissatisfied with their bodies at baseline displayed worsened body satisfaction [17,18] and more negative emotions [18] after being overtly exposed to media containing idealized physiques. This research has focused primarily on body satisfaction as the key moderator, and other possible body image variables have not been linked to gaze behaviors. For example, drawing from both social comparison [9] and self-objectification [11] theories, body appreciation (the tendency to accept and care for one’s body) and body-related envy (the tendency to desire to acquire the physical characteristics or achievements of another individual) have been shown to impact the relationship between idealized media exposure and body image [19,20,21]. Body appreciation may protect one from the potentially deleterious outcomes of exposure to physique-salient media [19,20] whereas envy may potentiate gaze behaviors and related negative outcomes [21]. However, the effects of these trait body image factors on voluntary exposure to, and influence of, physique media have not been studied.

Although less common than research involving media portraying *idealized* physiques, some studies have also examined the relationship between body image and exposure to *non*-*ideal* physique images [22,23,24,25,26,27]. Whereas exposure to idealized (muscular) men decreased men’s body satisfaction, exposure to media depicting average-sized men did not affect body satisfaction scores [28]. Furthermore, for vulnerable populations (e.g., women high in internalization, body dissatisfaction, neuroticism, or body anxiety), viewing average-sized or overweight models generally promoted healthier body ideals compared to viewing control images [25,26]. Viewing non-ideal physique images may even elicit positive affective outcomes for both men and women, such that body image improves in response to viewing these images [22,23,29,30]. Whereas the non-ideal female physique is consistently depicted and described as being overweight or heavy, the male non-ideal physique can diverge from the muscular ideal by being either thin or overweight [28,29]. Comparisons between exposure to idealized and non-idealized physique imagery among both men and women have not been reported. Furthermore, the majority of media exposure-related research has focused on explicit and specific exposure rather than examining the voluntary acquisition of physique imagery. In this way, it is less clear how men and women *choose* to consume (or avoid) physique-salient media in more naturalized environments, and the trait variables that might moderate such voluntary consumption of physique-salient media. People often choose what media they consume (e.g., decision to buy a magazine or to curate social media streams) and therefore understanding choice in physique-salient consumption is highly relevant for external validity. The current study was designed to investigate how men and women voluntarily looked at task-irrelevant images of same-sex models with idealized and non-idealized physiques. The primary aim was to elucidate the contributions of body-related envy and body appreciation towards indices of media consumption in more naturalized environments.

Whereas a variety of trait variables can moderate the effects of overt physique-salient media exposure on body image, these conclusions have been drawn from studies in which participants are explicitly exposed to and instructed to look at such images (e.g., [28,29]). Thus, the factors that influence individuals’ *voluntary* media consumption remain less clear. In addition, some studies suggest that individuals may react differently to physique-salient images due to differences in the way stimuli are processed and compared to their own body schema (e.g., body representation). Specifically, researchers theorize that an individual’s body image (for a review, see [31]) and propensity for social comparative emotions [32] may bias his/her processing of body-related stimuli (e.g., the prioritization of different parts of the body), resulting in individual differences in voluntary gaze patterns at physique-salient media. An individual’s appreciation of their own body and tendency for affective body-related social comparisons (e.g., envy) may shape voluntary gaze patterns. For example, body appreciation may lead to less gaze at media or may protect from the effects of media [20] whereas body-related envy may perpetuate greater viewing of ideal images that foster upward social comparisons [9]. These differences in gaze patterns, as well as the manner in which individuals compare themselves to an observed body, may help to explain why some individuals react more strongly than others to physique-salient media [33,34].

To our knowledge, biases toward physique-salient media have been focused on body (dis)satisfaction as an individual characteristic that might impact viewing patterns and outcomes (e.g., [31,35,36]), but individuals with high and low trait levels of body appreciation have not been studied. This observation is important because there is some evidence to suggest that these individuals might choose to consume physique-salient media differently. In fact, body appreciation is a unique body image factor compared to body satisfaction that is specific to valuing one’s body and orienting cognitive processing to protect a positive view of the body [20]. For example, women who have higher body appreciation may purposefully avoid consuming idealized media [19,37], but show indifference towards non-idealized media [19]. Furthermore, body-related envy is understudied yet may be an important individual factor linked to physique-salient media viewing. Specifically, men and women with high levels of trait body envy may compare their bodies to others’ bodies more frequently than non-envious individuals [21]. These more frequent comparisons may result in more frequent or longer voluntary gaze at physique-salient media [38]. This empirical evidence is also supported within theoretical tenets of social comparison and self-objectification theories [9,11]. As such, the purpose of the present study was to assess the relationship between individuals’ body image-related trait variables (e.g., body appreciation and body envy) and voluntary media consumption of idealized and non-idealized physiques in a relatively naturalized environment. A secondary aim was to examine voluntary media consumption behaviors as correlates of changes in affect following media exposure.

The current study advances previous literature on biases toward physique-salient media in four important ways. First, two previously unexamined trait variables—body appreciation and body envy—that may affect processing of physique-salient stimuli were considered. Second, the study design was based on the best practices for body image research [6] and in more naturalistic experimental settings [39]: using a cover story, including a distractor task, and using non-idealized physiques as control images. Third, the study population includes both men and women. The inclusion of men is important because the majority of research in this area focused on women [36,40,41] and the effects for women cannot be assumed to generalize to men [6]. Fourth, hidden cameras were used to covertly monitor gaze as opposed to the eye-tracking headgear used in similar studies to overtly monitor gaze [35,36,40,41]. Eye-tracking headgear creates an implied social presence and/or overt knowledge of an experimenter monitoring system, and can dramatically affect whether or not participants choose to look at physique-salient stimuli [42]. Indeed, previous research has shown that participants execute fewer spontaneous gaze shifts to physique-salient images when overt gaze recording systems, such as eye trackers, are used compared to when gaze is covertly tracked via a hidden camera [42,43]. As such, a covert gaze tracking protocol was used in the present study in an attempt to record more natural gaze patterns and to better conceal the true purpose of the research. A similar protocol has been used successfully to measure gaze in previous research (e.g., [42]).

Based on social comparison [9] and self-objectification theory [11] theories as well as previous literature demonstrating similar consequences of viewing idealized and non-idealized physique images for men and women (despite effect sizes being smaller in men), the following predictions were formed. First, body envy will be positively associated with the number of looks and time spent gazing at idealized, but not average-weight, physiques. Second, body appreciation will be (a) negatively associated with gaze at idealized physiques, and (b) positively associated with gaze at average-weight physiques. Third, gaze at idealized physiques will be associated with (a) increased negative affect, and (b) decreased positive affect, from first exposure to the end of the study. The inverse was predicted for the association between affect and exposure to average-weight physique images.

## 2. Methods

### 2.1. Participants

Participants were recruited from the University of Toronto community through classes and posters advertising the study. A total of 143 participants were recruited for the study. After the study debrief, two participants requested that their recordings not be analyzed leaving a sample of 141 (women: *n* = 89; men: *n* = 52). Participants were between the ages of 18 to 35 years old, had normal or corrected vision, and were naïve to the purpose of the study prior to and during data collection. All participants provided informed consent prior to completing the study and again at the end of the study after they were debriefed and were informed that they were covertly filmed in the testing room. Participants received financial compensation for their participation. The procedures were reviewed and approved by the university’s Research Ethics Board.

### 2.2. Procedure

The experiment took place in a testing room in a laboratory on the University of Toronto campus. The testing room was staged to look like a room that was transitioning from a testing room to a graduate student office (similar to [42]). The testing room (office) featured three desks. Two matching large “student” desks were situated against the left and right walls of the room and one small “testing” desk was placed in the middle of the wall opposite the door. The desks on the left and right of the central testing desk resembled disorganized graduate student desks with academic papers, files, computers, shoes, etc. on and surrounding the desks. Critically, in addition to the clutter, each desk had one 22 cm × 28 cm calendar featuring an image of an idealized- or an average-physique model. The calendar showing the idealized model was on one desk and the calendar showing the average-physique model was on the other desk. The position of the calendars was matched between the two desks (near the back of the desk approximately 30 cm from the side) and the location of the idealized and average-physique model on the desk to the left or right of the participant was randomized.

When the participants arrived for the testing session, they were told a general cover story that they would be completing a study that examined “how people perceive and process images.” Thus, from the participants’ perspective, the main purpose and method of the study was to complete a computer-based task that explored perceptions of visual stimuli. Upon entering the testing room, the researcher covertly directed the participants’ gaze to each of the staged “student” desks with the calendars on them by saying, “Sorry it’s so messy in here, they’re letting us use the room, but they will be moving graduate students in here and here.” The researcher pointed to the desks when they indicated each location, but not to any specific item on or around any of the desks. These specific instructions were used to prime the participant to look at each desk to increase their potential awareness of the two physique-salient images without specifically directing their focus to the calendars and the images. Participants were then seated at the “testing” desk (between the two “student” desks with the calendars) and completed the following tasks in this specific order: (1) informed consent; (2) a measure of affect; (3) a computer task; (4) a post-test questionnaire including trait measures of body appreciation, body-related envy, and physical self-perceptions attractiveness, thinness, and physical strength, as well as another measure of affect; and (5) a debriefing regarding the true purpose of the experiment at the end of the study.

The computer task (in part 3) took approximately 20 min to complete and included a one-minute rest period at the halfway point. During the task, participants completed an Affect Misattribution Procedure (AMP) paradigm, which uses implicit procedures to measure stereotype endorsement [44]. These data are reported elsewhere [45]. Participants’ viewing patterns during the one-minute rest period were isolated as the focus of this experiment because they represented the key indices of spontaneous media consumption—specifically, how often and for how long each individual chose to look at the calendars featuring idealized- and average-physique bodies when they had nothing else to do during a rest period (see [42]).

Each calendar depicted only one physique image and the images were sex-matched to the participants (i.e., calendars featuring male models were placed in the room when men completed the task and calendars featuring female models were placed in the room when women completed the task). The calendars were placed equidistant from the participant (approximately 110 cm away) and directly to the left and right of the central testing desk where the participant sat. Each image included one model wearing a black swimsuit (shorts for males, bikini for females) so that the majority of the model’s body was unclothed and visible to the viewer. The model in the idealized-physique image shown to men was of a muscular male, whereas the model in the idealized-physique image shown to women was of a thin female. Both the male and female average-physique model appeared to be at the upper end of healthy body mass indices, bordering on overweight. We considered the slightly overweight models to represent the Canadian average, given that 63% of Canadians 18 years and over self-report body mass indices in the overweight or obese category [46]. All models were Caucasian. These images were pilot tested on an unrelated convenience sample to determine how attractive and thin/overweight each model appeared. These pilot test findings are presented in the Results section. To control for any left/right looking bias as a potential confound in the present study, we randomized the student desk (i.e., left or right) on which the calendars with the idealized- and average-physique models were placed.

One pinhole camera (Miniature Pinhole CCD Color Camera 420TVL 3.6 mm, Sony, Tokyo, Japan) was hidden in each of two different objects (tissue paper box on the right desk and tea canister on the left desk) beside each calendar on the student desks (similar to [42]). Although participants were informed that all their responses and behavior were going to be recorded when they were in the testing room, participants were unaware of the precise location of the cameras hidden next to each calendar that recorded how often the participants glanced at the calendar images. At the end of the study, participants were asked if they noticed anything odd about the experimental procedure; no participant mentioned noticing the hidden cameras. Participants were informed of the full nature of the study and of the hidden cameras during the debriefing at the end of the testing session. At that point, participants were asked if they consented to the use of their video data and were given the opportunity to withdraw from the study. Two participants requested that their recordings not be analyzed after the debriefing. All other participants consented to keeping their video data in the study.

### 2.3. Measures

#### 2.3.1. Demographics

Participants reported their sex, age, ethnicity, education, and primary language spoken at home. Physical activity was a behavior thought to be important for physique shape and size and was assessed with the International Physical Activity Questionnaire [47]. Scores for the number of days in the last week and minutes engaged in moderate and vigorous activity were computed based on standards [47]. Participant scores were dichotomously coded as meeting or not meeting the recommended minimum of 150 min of moderate-vigorous physical activity per week [48]. Participants also provided physical self-perceptions [49] of attractiveness (*I am attractive*), thinness (*I am thin*), and physical strength (*I am physically strong*) on separate 7-point Likert scales anchored at 1 (*not at all*) and 7 (*very much*) to assess participants’ current beliefs of their physique-related features. These items were included as potential covariates in the analysis. See Table 1 and Table 2 for details.

#### 2.3.2. Body Appreciation

The Body Appreciation Scale (BAS; [50]) was used to measure qualities of positive body image. The scale consists of 13 items (e.g., *Despite its flaws, I accept my body for what it is*) scored on a 5-point Likert scale from 1 (*never*) to 5 (*always*). The mean of the items was calculated with higher scores indicating greater body appreciation. In the current study, the Cronbach’s alpha internal consistency coefficient of the BAS items was α = 0.91.

#### 2.3.3. Body-Related Envy

The Dispositional Body-related Envy Scale (DBES; [21]) is an 8-item scale used to assess feelings of body-related envy. Participants responded to the items (e.g., *When it comes to thoughts about my body/physique, I feel envy every day*) on a 5-point Likert scale from 1 (*strongly disagree*) to 5 (*strongly agree*). The mean of all items was calculated, with higher scores representing higher body-related envy [21]. The internal consistency for the items from the DBES in the current study was α = 0.92.

#### 2.3.4. Affect

Affect was measured using the Positive and Negative Affect Schedule (PANAS; [51]). The scale consists of 20 items measuring positive (*n* = 10) and negative (*n* = 10) affect. Participants responded by indicating the extent to which they felt each emotion “at the present moment” on a scale from 1 (*very slightly or not at all*) to 5 (*extremely*), with higher scores indicating greater positive and negative affect. The PANAS was completed by participants after they were provided with the cover story regarding the room transitioning from lab to office and then again at the end of the study. To assess change in affect over time, a change score of post-test minus pre-test was calculated. The PANAS items exhibited good internal consistency at both timepoints, with respect to both positive, α = 0.85/0.90, and negative affect, α = 0.82/0.84.

#### 2.3.5. Media Consumption Behaviors

Three research assistants, who were blinded to the purpose of the experiment as well as to the nature and characteristics of the physique images near the cameras, viewed the videos recorded from both cameras. The research assistants recorded the number of times participants looked at each camera and the duration of each gaze (in ms). Each look was identified as direct eye contact with the camera/image. The duration of each look was recorded from the time that participants stared into the camera until a shift in eye gaze was observed. Only data from the one-minute wait period in the middle of the AMP computer task were considered for in-depth analysis (see [42,43]). Any discrepancies in coding between the research assistants were reviewed and discussed until consensus was reached. Based on these procedures, first look (which physique image was looked at first), the number of looks at each of the media, and the amount of time spent looking at the ideal- and average-physique images were three key outcomes of this study.

### 2.4. Data Analysis

Preliminary analyses included the calculation of means and standard deviations, frequency counts, and Pearson and Spearman correlation coefficients. Analysis of variance was used to explore differences between men and women on the gaze behaviors, and Bonferroni-corrected *t*-tests were conducted to explore differences on the main study variables for men and women, and within-group differences on gaze behavior comparisons.

The main analyses involved logistic and linear regression models to determine whether measures of body image were correlated with which physique-image participants would look at first, the number of looks, and the amount of time spent looking at the ideal- and average-physique images. The models were run separately for men and women because the media were sex-specific images. Due to evidence in the present data of multicollinearity between body appreciation and dispositional body-related envy, separate regressions were run for each body image variable, adjusting for participants’ self-perceptions of attractiveness, thinness, and physical strength. Correlations and linear regressions were also used to address the secondary aim of this study by assessing voluntary exposure to the physique-salient images and changes in positive and negative affect.

All statistical analyses were conducted using SPSS (Version 26). Alpha was set at 0.05, unless otherwise noted. Effect size calculations and exact *p* values are reported where relevant.

## 3. Results

### 3.1. Pilot Study

A pilot study was conducted to explore perceptions of the models used for the idealized- and average-physique images by same-sex viewers in terms of body size and attractiveness. A convenience sample (*N* = 18; 9 women) rated the models’ body size on a scale from 1 (*thin*) through 4 (*average*) to 7 (*overweight*), and the models’ attractiveness on a scale from 1 (*unattractive*) to 7 (*attractive*). None of the individuals in this convenience sample participated in the main study. Men perceived the average-physique male model to be more average weight (*M* = 4.33, *SD* = 1.00) than the ideal-physique male model (*M* = 3.11, *SD* = 0.93), *t*(8) = 5.50, *p* = 0.001, *d*_z_ = 1.83, but there was no significant difference in the perceived attractiveness of the average-physique model (*M* = 3.78, *SD* = 0.97) compared to the ideal-physique model (*M* = 4.56, *SD* = 1.01), *t*(8) = 2.14, *p* = 0.065, *d*_z_ = 0.71. Similarly, women perceived the average-physique female model to be more average weight (*M* = 4.78, *SD* = 0.44) than the ideal-physique female model (*M* = 1.78, *SD* = 0.83), *t*(8) = 12.73, *p* < 0.001, *d*_z_ = 4.24, but again there was no significant difference in the perceived attractiveness of the average-physique model (*M* = 5.22, *SD* = 1.39) compared to the ideal-physique model (*M* = 5.00, *SD* = 1.32), *t*(8) = 0.36, *p* = 0.73, *d*_z_ = 0.12. Thus, for both men and women, the ideal- and average-physique models were not different with respect to attractiveness and differed primarily in terms of body size.

### 3.2. Preliminary Analyses

#### 3.2.1. Descriptives

Seven participants were excluded due to technical issues (*n* = 3), for using their cell phone during testing (*n* = 1), for touching a hidden camera (*n* = 1), or for interacting with the physique-salient images (e.g., flipping through the calendar on which a physique image was presented; *n* = 2). Thus, the final sample consisted of 134 participants (65% women). Participants ranged in age from 18 to 31 years old (*M* = 19.7 years, *SD* = 1.5) and most participants self-identified as Caucasian (50%) or Chinese (15%) and spoke English at home (75%).

Descriptive statistics and correlations between variables are reported in Table 1 and Table 2. For descriptive purposes, 81% of the men and 67% of the women in the sample reported engaging in at least 150 min of moderate-to-vigorous physical activity per week which reflects a level of activity conducive to meeting Canadian guidelines. On average, participants rated their perceptions of thinness, strength, and attractiveness near the mid-point of the scales (i.e., average ratings between 4 and 5 on a 7-point Likert type scale). There were no differences between men and women’s reporting of attractiveness and thinness, *t*(132) = 0.45, *p* = 0.66, *d* = 0.083 and *t*(75.46) = 0.19, *p* = 0.85, *d* = 0.037, respectively; however men reported significantly higher perceptions of strength compared to women, *t*(132) = 1.91, *p* = 0.058, *d* = 0.35. See Table 1 and Table 2 for details. Furthermore, men reported significantly lower body envy scores, *t*(132) = 2.39, *p* = 0.018, *d* = 0.44, compared to women. There were no differences on body appreciation scores, *t*(132) = 0.41, *p* = 0.68, *d* = 0.073. There were no differences (*p*’s = 0.08 to 0.59) between men and women’s ratings of positive or negative affect at first exposure or following media consumption.

#### 3.2.2. Gaze towards the Physique-Salient Media

To assess voluntary media consumption behaviors, separate 2 (Sex: men, women) × 2 (Model-physique: ideal, average) mixed measures ANOVAs were conducted on the number of looks and look duration. There were no significant main effects or interactions with respect to the number of looks towards the models (*F*s < 1). Participants tended to look for longer at the ideal-physique model than the average-physique model, *F*(1, 132) = 3.51, *p* = 0.063, η^2^ = 0.026, and there were no sex-specific effects or interactions (*F*s < 1).

Men looked at the ideal-physique image first 51% of the time, compared to looking first at the average-physique image first (34%) or not spontaneously looking at either image (15%). Women looked at the ideal-physique image first 47% of the time, compared to looking first at the average-physique image (43%) or not choosing to look at either image (10%). There were no differences in gaze towards the ideal- vs. average-physique model in terms of the number of looks for men, *t*(46) = 0.52, *p* = 0.61, *d*_z_ = 0.076, or women, *t*(86) = 0.18, *p* = 0.86, *d*_z_ = 0.019. No differences were observed for look duration for men, *t*(46) = 0.99, *p* = 0.33, *d*_z_ = 0.14, or women, *t*(86) = 1.17, *p* = 0.25, *d*_z_ = 0.13.

### 3.3. Main Analyses

#### 3.3.1. Correlates of Voluntary Media Consumption.

##### Body Image Variables Did Not Correlate with First Look

Separate logistic regressions were performed to explore the effects of body-related envy and body appreciation on the likelihood that participants chose to look at the ideal-physique model first, adjusting for self-perceptions of attractiveness, thinness, and physical strength. Neither body-related envy nor body appreciation were significantly correlated with whether men or women chose to look at the ideal-physique or average-physique model first.

##### Body Image Variables Did Not Correlate with Gaze towards the Ideal-Physique Image

Linear regressions assessing the number of looks and gaze duration at the physique-salient images from body-related envy and body appreciation, when adjusting for self-perceptions of attractiveness, thinness, and physical strength, were generally consistent with correlations (Table 1 and Table 2) for both men and women. For men and women, body-related envy did not correlate with the number of looks (*R*^2^ = 0.02 and 0.06, respectively), nor how long men and women looked at the ideal-physique image (*R*^2^ = 0.16 and 0.02, respectively). Similarly, body appreciation did not correlate with how often (*R*^2^ = 0.01 and 0.06), nor for how long they looked at the ideal-physique image (*R*^2^ = 0.14 and 0.01) for men and women, respectively.

##### Body Image Variables as Correlates of How Often Men Looked at the Average-Physique Image

For men, body envy (*ß* = 0.50, *p* = 0.006) was significantly correlated with the number of looks at the average-physique model, *R*^2^ = 0.19. In a separate model, body appreciation was also a significant correlate, *ß* = −0.49, *p* = 0.009, *R*^2^ = 0.19, such that lower body appreciation was also associated with more frequent looks at the average-physique model. The models assessing time spent gazing at the average-physique model were not significant.

For women, body envy did not correlate with the number of looks at the average-physique model, *R*^2^ = 0.02, nor overall gaze time, *R*^2^ = 0.04. Body appreciation was also not significantly related to the number of looks, *R*^2^ = 0.01, *p* = 0.89, nor the time spent looking at the average-physique image, *R*^2^ = 0.03, *p* = 0.61.

#### 3.3.2. Mood Changes from First Exposure to Post-Exposure

##### Participants Experienced a General Affective Decrease over Time

Men reported fewer positive emotions following exposure to the physique-salient images, *t*(38) = 2.00, *p* = 0.053, *d*_z_ = 0.32, but there was no significant change in negative affect, *t*(38) = 0.62, *p* = 0.54, *d*_z_ = 0.10. Women also reported fewer positive emotions at post-test compared to first exposure, *t*(85) = 3.34, *p* = 0.001, *d*_z_ = 0.36, as well as fewer negative emotions, *t*(85) = 2.07, *p* = 0.042, *d*_z_ = 0.22. These findings suggest that participants experienced a general affective decrease over the course of the study.

##### Voluntary Media Consumption Did Not Correlate with Change in Positive Affect

Linear regressions were conducted to determine how participants’ voluntary exposure to the ideal- and average-physique images was correlated with changes in positive and negative affect, adjusting for participants’ physical self-perceptions (perceived attractiveness, thinness, and physical strength). For the ideal- and average-physique images separately, the exposure variables included in the model were the number of looks and the time spent looking at the image. See Table 3 for details.

Exposure to the ideal-physique model did not correlate with change in positive affect for men, *F*(5, 32) = 1.15, *p* = 0.35, *R*^2^ = 0.15. The model for women was significant, *F*(5, 84) = 3.24, *p* = 0.010, *R*^2^ = 0.17, with perceptions of strength emerging as the only significant correlate. Gaze at the average-physique model also did not correlate with change in positive affect for men, *F*(5, 32) = 1.22, *p* = 0.32, *R*^2^ = 0.16, or women, *F*(5, 79) = 1.95, *p* = 0.095, *R*^2^ = 0.11.

##### Voluntary Media Consumption Was Correlated with Change in Negative Affect for Men

Men’s change in negative affect was correlated with their voluntary exposure to the ideal-physique model, *F*(5, 32) = 5.96, *p* = 0.001. Specifically, time spent looking at the ideal-physique image (*ß* = -.42) was a significant correlate of change in negative affect such that more time spent looking at the ideal-physique model was associated with lower values of negative affect post-exposure relative to pre-exposure (a decrease in negative affect), and less time spent looking at the ideal-physique was associated with higher values of negative affect in the post-exposure phase than in the pre-exposure phase (an increase in negative affect). Women’s change in negative affect was not related to voluntary exposure to the ideal-physique image, *F*(5, 79) = 1.71, *p* = 0.14, *R*^2^ = 0.10, or the average-physique image, *F*(5, 79) = 1.78, *p* = 0.13, *R*^2^ = 0.10. See Table 3 for details.

## 4. Discussion

The present experiment examined whether body image variables influence how individuals choose to consume physique-salient media, as well as how media consumption behaviors subsequently influence affect. As a summary of the main findings for men, higher levels of body-related envy and lower levels of body appreciation were associated with more frequent looks at the average physique, while time spent looking at the ideal physique was associated with a reduction in negative affect. For women, the current body image variables of interest were not significantly related to voluntary media consumption. Overall, these data indicate that body image variables can underpin how individuals choose to expose themselves to physique-salient media, particularly for men, and that media consumption may have consequences for post-exposure affect. The implications of these findings will be addressed in turn.

First, it is important to address participants’ overall voluntary consumption of the physique-salient images. During the one-minute rest period, men/women glanced an average of 1.9/1.8 times at the ideal- and 1.8/1.9 times at the average-physique image. On average, men/women spent 8.7/9.5 s spent gazing at either image. Although the time looking at the images may seem low, it accounted for approximately 15% of the total time period measured. Recall also that the images were discretely placed in positions that were perpendicular to participants and so some effort was required by the participant to gaze at them (i.e., a change in gaze that involved a head turn). Furthermore, although findings are mixed [6], when exposure duration moderates the effect of media exposure on body image, the relationship is such that shorter exposures have a stronger effect (for a meta-analysis, see [16]). Based on these findings [16], participants’ exposure to the images in the current experiment was sufficient to produce a change in body image.

With respect to how body image impacted voluntary media consumption, the measured trait variables appear to be stronger correlates of behavior in men than women. Indeed, neither body-related envy nor body appreciation were significantly correlated with women’s media consumption behaviors. It is possible that women in this study did not feel similar enough to the models displayed in the calendar to feel envious of them. Previous research suggests that envy is more likely to occur when individuals compare themselves to similar others [52,53]. For instance, college students are more likely to compare their bodies to the bodies of other college students, rather than to the bodies of models or athletes [54]. Therefore, the female models presented on the calendars may not have represented a salient social comparison for the current sample, and as a corollary effect, media consumption behaviors were not related to dispositional body-related envy. Alternatively, it is possible that our data provide a more veridical measurement of participants’ voluntary media consumption because tasks in previous research required participants to look at images, whereas participants in the present study were able to volitionally consume the physique-salient media, and thus may be more reflective of people’s natural behaviors.

In contrast, men’s trait levels of body-related envy and body appreciation were significantly correlated with some aspects of their voluntary media consumption. Counter to our hypotheses, higher body envy and lower body appreciation were associated with looking more frequently at the average-physique image. Participants with relatively high body envy and low body appreciation preferentially exposed themselves to the average-physique image, which aligns with previous literature showing that average-physique images do not pose the same threat to body positivity as idealized images [25]. It should be noted, however, that the model used for the average-physique image was dressed in a revealing manner (i.e., wearing only a swimsuit) and was perceived to be as attractive as the ideal-physique model in pilot testing. Because individuals high in positive body image report avoiding any images that threaten their positive body image [55], it is possible that the average-physique model used in the present study met these subjective criteria for a “threatening” stimulus, which possibly led men high on body appreciation to also avoid gazing at this image. This finding, if confirmed in future studies, provides further evidence on the possible protective nature of body appreciation that may extend beyond the effects of body satisfaction [20].

Finally, declines in positive and negative affect over the course of the experiment suggest participants experienced a general dampening of emotions, perhaps due to the repetitive nature of the computer task. For men, however, this reduction in negative affect was correlated with the time they spent looking at the ideal-physique image such that more time spent looking at the ideal-physique was associated with lower negative affect. It is possible that the current sample of men felt they compared positively to the ideal-physique model, particularly considering that over 80% of the sample reported meeting moderate-to-vigorous physical activity guidelines. If this is the case, then the ideal-physique model might actually have acted to protect against increases in negative affect. For women, time spent looking at the ideal-physique image was related to a decrease in positive emotions, although the regression model was not significant. Nevertheless, the negative relationship between the time women spent looking at the ideal-physique image and positive affect still provides some insight into how the processing of physique images influences emotions. Whereas increases in negative affect are thought to lead to unpleasant emotions such as anxiety and anger, decreases in positive affect are thought to lead to sadness and discouragement [51]. Thus, women who gazed for longer at the idealized image may have felt more sadness and discouragement about their bodies in comparison to the idealized body. Of note, women did not experience a significant change in affect related to viewing the average-physique model. This finding is not consistent with previous findings of greater positive affect in women viewing healthy weight versus thin models [25]. This affective change seen in previous research, however, was limited to a sample of women with high baseline anxiety [25], a trait which was not measured in the present experiment. Future research should examine additional trait variables to further explore moderators of the relationship between average-physique media consumption and affect.

### Limitations

There may be limits to the generalizability of the current study to the broader population, given the convenience sample of volunteer participants. That is, the sample was drawn from a university-aged population who may hold different views of their bodies than older and younger men and women from the broader population [56]. Furthermore, the sample was highly diverse in terms of ethnicity, and people of different ethnicities may have varying ideals pertaining to what constitutes an attractive body shape. Of note, although the use of both idealized and non-idealized physique images has been recommended (e.g., [6]), it is possible that participants’ exposure to both physique types could have created a counteractive effect. For example, if a woman with low body satisfaction first looked at the idealized image and felt poorly, and subsequently looked at the average physique and felt better about her appearance, such physique-specific influences on affect would not be captured by the current design. Furthermore, although the experimental conditions (wherein participants were exposed to task-irrelevant physique-salient images) may be different than some instances of media consumption, such as scrolling through social media online, participants’ voluntary gaze shifts to and examination of the calendars serve as a proxy for media consumption. The conditions of the present study may be particularly representative of scenarios where individuals are exposed to a variety of physique-salient images and can preferentially look at or avoid certain images, such as when choosing between magazines at a store or examining different advertisements displayed on a wall or in a magazine. Finally, the use of hidden cameras to assess gaze behaviors could be viewed both as a strength and potential weakness of the current study. Although using the covert video recording technique enabled the observation and analysis of spontaneous gaze behavior, it is perhaps less precise than eye-tracking technology that provides more spatially accurate recordings of where observers fixated on the image (e.g., model’s face, stomach). Given evidence that eye-tracking hardware changes participants’ gaze patterns in a manner that reflects impression management [42], the authors felt that it was more effective and appropriate to trade precise but overt measures of gaze with eye-tracking technology for ecological validity associated with covert gaze recoding.

## 5. Conclusions

The purpose of the present study was to understand body-image factors related to physique-salient media consumption. Men and women did not differ overall in their spontaneous gaze behaviors towards physique-salient images, but indices of media consumption were partially mediated by trait variables. Whereas body image factors were not related to media consumption behaviors in women, body envy was positively correlated, and body appreciation was negatively correlated with men’s voluntary exposure to average-physique media. These sex differences potentially reflect women’s over-exposure to a range of physique-salient media, compared to men’s less frequent exposure to physique-salient media and in particular to average-physique images. Overall, the results of this study are broadly consistent with social comparison [9] and self-objectification [11] theories and contribute to a growing literature on body image by providing insight into the trait variables associated with men and women’s media consumption, along with ecologically valid measures of how these the media consumption preferences manifest (i.e., through voluntary gaze behaviors). Furthermore, this research provides preliminary support for a mechanism (i.e., selective exposure) by which individuals manage the emotional experience that co-occurs with physique-related media consumption. Finally, on the methodological level, the present experiment represents a novel methodology for assessing voluntary media consumption and the influences that this consumption has on affect with greater ecologically validity than previous methods.

## Figures and Tables

**Table 1:** Descriptive statistics and correlations between study variables for women.

Variable	M (SD)	1	2	3	4	5	6	7	8	9	10	11	12	13	14	15	16	17	18
1. Age	19.51 (1.03)	--	--	--	--	--	--	--	--	--	--	--	--	--	--	--	--	--	--
2. Physical activity ^1^	--	−0.001	--	--	--	--	--	--	--	--	--	--	--	--	--	--	--	--	--
3. Caucasian ^1^	--	0.2	0.28 **	--	--	--	--	--	--	--	--	--	--	--	--	--	--	--	--
4. Attractiveness	4.49 (1.33)	0.078	−0.028	0.05	--	--	--	--	--	--	--	--	--	--	--	--	--	--	--
5. Thinness	4.23 (1.40)	0.095	−0.15	0.1	0.41 **	--	--	--	--	--	--	--	--	--	--	--	--	--	--
6. Physical strength	4.58 (1.19)	0.17	0.33 **	0.22 *	0.22 *	0.098	--	--	--	--	--	--	--	--	--	--	--	--	--
7. Body appreciation	3.71 (0.63)	−0.065	−0.01	0.098	0.52 **	0.51 **	0.25 *	--	--	--	--	--	--	--	--	--	--	--	--
8. Envy	2.62 (0.94)	0.14	0	0.011	−0.35 **	−0.41 **	−0.2	−0.70 **	--	--	--	--	--	--	--	--	--	--	--
9. Number of looks at ideal	1.83 (1.34)	−0.012	0.039	−0.099	−0.03	−0.006	−0.13	−0.039	−0.024	--	--	--	--	--	--	--	--	--	--
10. Number of looks at average	1.85 (1.37)	0.054	0.042	−0.041	−0.016	−0.007	−0.12	−0.053	0.085	0.61 **	--	--	--	--	--	--	--	--	--
11. Time looking at ideal	5.06 s (5.03)	−0.002	0.02	−0.038	0.004	0.041	0.083	0.013	−0.11	0.65 **	0.23 *	--	--	--	--	--	--	--	--
12. Time looking at average	4.40 s (4.15)	0.02	−0.052	−0.005	0.023	0.12	−0.13	0.033	0.038	0.43 **	0.68 **	0.35 **	--	--	--	--	--	--	--
13. Positive affect (first exposure) ^2^	2.71 (0.65)	0.07	0.049	0.22 *	0.30 **	0.11	0.22 *	0.17	0.052	−0.05	−0.073	0.022	0.071	--	--	--	--	--	--
14. Negative affect (first exposure)	1.39 (0.42)	−0.077	0.052	−0.074	−0.19	0.079	−0.24 *	−0.16	0.1	0.044	0.11	−0.14	0.097	0.086	--	--	--	--	--
15. Positive affect (post experiment)	2.57 (0.76)	0.11	0.14	0.24 *	0.17	0.062	0.30 **	0.17	0.025	0.05	−0.071	0.14	0.027	0.88 **	0.095	--	--	--	--
16. Negative affect (post experiment)	1.34 (0.39)	−0.036	0.1	−0.02	−0.25 *	0.012	−0.11	−0.22 *	0.14	−0.027	0.074	−0.15	0.096	0.073	0.85 **	0.035	--	--	--
17. Change in positive affect ^3^	−0.13 (0.37)	0.098	0.2	0.18	−0.16	−0.069	0.25 *	0.063	−0.049	0.2	−0.019	0.24 *	−0.063	0.046	0.045	0.52 *	−0.055	--	--
18. Change in negative affect ^3^	−0.050 (0.22)	0.082	0.13	0.082	−0.082	−0.13	0.25 *	−0.11	0.067	−0.13	−0.081	−0.01	−0.014	−0.031	−0.37 **	−0.12	0.17	−0.18	--

Notes. ^1^ Spearman’s rho reported for dichotomous variables. ^2^ Due to experimenter error, nine participants did not complete the initial PANAS assessment, reducing the sample size to 86 females for analyses involving affect following exposure to the physique-salient images. ^3^ Change scores calculated as (post experiment minus first exposure). * indicates the correlation is significant at the 0.05 level (2-tailed). ** indicates the correlation is significant at the 0.01 level (2-tailed).

**Table 2:** Descriptive statistics and correlations between study variables for men.

Variable	M (SD)	1	2	3	4	5	6	7	8	9	10	11	12	13	14	15	16	17	18
1. Age	20.17 (1.95)	--	--	--	--	--	--	--	--	--	--	--	--	--	--	--	--	--	--
2. Physical activity ^1^	--	0.13	--	--	--	--	--	--	--	--	--	--	--	--	--	--	--	--	--
3. Caucasian ^1^	--	0.095	0.029	--	--	--	--	--	--	--	--	--	--	--	--	--	--	--	--
4. Attractiveness	4.60 (1.10)	−0.048	0.26	0.23	--	--	--	--	--	--	--	--	--	--	--	--	--	--	--
5. Thinness	4.17 (1.78)	0.22	0.05	0.069	0.29 *	--	--	--	--	--	--	--	--	--	--	--	--	--	--
6. Physical strength	4.98 (1.13)	−0.038	0.13	0.065	0.24	−0.24	--	--	--	--	--	--	--	--	--	--	--	--	--
7. Body appreciation	3.75 (0.68)	−0.14	0.072	0.007	0.56 **	0.17	0.35 *	--	--	--	--	--	--	--	--	--	--	--	--
8. Envy	2.23 (0.85)	−0.006	0.1	−0.15	−0.58 **	−0.27	−0.14	−0.68 **	--	--	--	--	--	--	--	--	--	--	--
9. Number of looks at ideal	1.89 (1.56)	0.085	0.21	0.033	−0.14	−0.12	−0.038	−0.21	0.2	--	--	--	--	--	--	--	--	--	--
10. Number of looks at average	1.79 (1.43)	0.045	0.23	−0.083	0.083	−0.14	0.024	−0.25	0.29 *	0.56 **	--	--	--	--	--	--	--	--	--
11. Time looking at ideal	5.59 s (8.87)	−0.072	0.18	−0.025	−0.29 *	−0.22	0.13	−0.13	0.29	0.48 **	0.28	--	--	--	--	--	--	--	--
12. Time looking at average	3.96 s (3.95)	0.043	0.23	−0.02	−0.058	−0.17	−0.17	−0.18	0.26	0.47 **	0.65 **	0.21	--	--	--	--	--	--	--
13. Positive affect (first exposure) ^2^	2.77 (0.66)	0.03	0.084	0.07	−0.038	0.43 **	0.15	−20	−0.019	−0.16	−0.11	−0.001	0.014	--	--	--	--	--	--
14. Negative affect (first exposure)	1.50 (0.58)	0.14	−0.071	0.22	−0.17	−0.048	−0.054	−0.22	0.41 **	0.035	−0.007	0.29	0.16	0.14	--	--	--	--	--
15. Positive affect (post experiment)	2.65 (0.76)	0.085	0.13	0.034	−0.036	0.42 **	0.22	0.15	0.046	−0.051	−0.018	0.063	−0.004	0.83 **	0.072	--	--	--	--
16. Negative affect (post experiment)	1.50 (0.56)	−0.007	0.032	0.053	−0.001	−0.16	0.034	−0.18	0.25	−0.077	−0.2	−0.053	−0.01	−0.12	0.72 **	−0.001	--	--	--
17. Change in positive affect ^3^	−0.14 (0.43)	0.2	0.016	0.08	0.17	0.078	0.31	0.17	−0.035	0.11	0.18	0.14	0.054	−0.045	−0.091	0.52 **	−0.072	--	--
18. Change in negative affect ^3^	−0.041 (0.42)	−0.18	−0.18	−0.2	0.39 *	−0.24	0.16	0.3	−0.34 *	−0.11	−0.15	−0.42 **	−0.085	−0.35 *	−0.48 **	−0.28	0.26	0.036	--

Notes. ^1^ Spearman’s rho reported for dichotomous variables. ^2^ Due to experimenter error, nine participants did not complete the initial PANAS assessment, reducing the sample size to 39 males for analyses involving affect following exposure to the physique-salient images. ^3^ Change scores calculated as (post experiment minus first exposure). * indicates the correlation is significant at the 0.05 level (2-tailed). ** indicates the correlation is significant at the 0.01 level (2-tailed).

**Table 3:** Summaries of linear regression models assessing change in men and women’s positive and negative affect from physical self-perceptions of attractiveness, thinness, and physical strength (step 1), and voluntary consumption of idealized-physique and non-idealized physique media (step 2).

	Men				Women			
	Idealized Physique		Non-Idealized Physique		Idealized Physique		Non-Idealized Physique	
	b (SE)	*R* ^2^	b (SE)	*R* ^2^	b (SE)	*R* ^2^	b (SE)	*R* ^2^
Positive Affect Change								
Step 1		0.12		0.12		0.11		0.11
Attractive	0.031 (0.078)		0.031 (0.078)		−0.060 (0.032)		−0.060 (0.032)	
Thin	0.030 (0.045)		0.030 (0.045)		−0.002 (0.030)		−0.002 (0.030)	
Strong	0.11 (0.066)		0.11 (0.066)		0.093 ** (0.034)		0.093 ** (0.034)	
Step 2		0.15		0.16		0.17		0.11
Number of looks	0.029 (0.051)		0.058 (0.066)		0.041 (0.038)		0.012 (0.039)	
Gaze duration	0.54 (0.90)		0.41 (2.27)		0.91 (1.01)		−0.43 (1.32)	
Negative Affect Change								
Step 1		0.35		0.35		0.087		0.087
Attractive	0.25 ** (0.066)		0.25 ** (0.066)		−0.015 (0.020)		−0.015 (0.020)	
Thin	−0.12 ** (0.038)		−0.12 ** (0.038)		−0.019 (0.018)		−0.019 (0.018)	
Strong	−0.045 (0.056)		−0.045 (0.056)		0.051 *(0.021)		0.051 * (0.021)	
Step 2		0.48		0.42		0.098		0.10
Number of looks	0.008 (0.039)		−0.088 (0.054)		−0.023 (0.024)		−0.024 (0.024)	
Gaze duration	−1.84 * (0.69)		0.10 (1.85)		0.32 (0.64)		0.82 (0.80)	

* indicates the coefficient is significant at the 0.05 level. ** indicates the coefficient is significant at the 0.01 level.

## Data Availability

Data is contained within the article.
